# Observations on Excision of the Superior Maxillary Bone; Illustrated by Seven Cases

**Published:** 1852-09

**Authors:** S. D. Gross

**Affiliations:** Professor of Surgery in the Medical Department of the University of Louisville; Louisville


### WESTERN JOURNAL

OF

### MEDICINE AND SURGERY.

SEPTEMBER, 1852.

---

ART. I.—*Observations on Excision of the Superior Maxillary Bone; Illustrated by seven Cases.* By S. D. GROSS, M. D., Professor of Surgery in the Medical Department of the University of Louisville.

The diseases of the superior maxillary bone, requiring the operation of excision, and generally described under the vague and unmeaning name of *osteo-sarcoma*, constitute a numerous and important group of affections, defying every attempt at correct classification. That this declaration is not a mere assumption, unsupported by facts, the writings of pathologists and surgeons abundantly attest. Much as has been written upon this subject within the last quarter of a century, there is still room for observation, both as it respects the nature, diagnosis, and treatment of these maladies. More light must be elicited before this branch of surgery can be placed upon a sure and solid foundation in relation to the propriety or impropriety of the operation of excision in any particular case, or in any particular class of diseases.

It is not my design, in this paper, to write a learned and elaborate article upon these affections; on the contrary, all I shall aim to do is, to point out a few of the more common and important, those which I have most frequently witnessed in practice, and those with which I am, consequently, most familiar from personal observation.

1.—By far the most frequent disease of the upper jaw is *encephaloid*, osteocephaloma, or soft cancer, which occurs here, as elsewhere, in both sexes, in all classes of individuals, and at all periods of life. I have seen it in young children under five years, at middle life, in old age, and in decrepitude. I am satisfied, however, that it is most frequent between the twentieth and fortieth years. In one of my patients it occurred at the age of sixty-six.

It is not known what influence, if any, is exerted upon the development of this disease by *occupation*, temperament, climate, and other circumstances. In every instance of it that has fallen under my observation, the tumor sprang up without any assignable, or palpable cause. Cases have been recorded in which it was apparently awakened by external injury, as a blow, the pressure of the pullikin, or fracture of the bone.

Encephaloid usually *begins* in the cavity of the antrum of Highmore, in connection with the mucous lining, or internal periosteum. Occasionally it takes its rise in the cancellated structure of the bone, in the socket of one of the teeth, in the gum, or in the periosteum, properly so called. In the first case, it generally progresses, with more or less rapidity, until it fills up the whole sinus, after which it encroaches upon the bony parietes of the cavity, pushing them out in every direction, and thereby pressing them against the surrounding structures. As the external wall is extremely thin, in fact, a mere shell, in the natural state, the morbid growth generally advances more rapidly in this direction than in any other, forming thus frequently, even at an early stage, quite a large tumor beneath the integuments of the cheek. By and by, as it proceeds in its development, it extends inwards towards the nostril, partially, sometimes wholly, occluding the corresponding cavity; upwards toward the floor of the orbit, compressing, and ultimately protruding the ball of the eye; downwards towards the roof of the mouth, displacing the tongue, and diminishing the buccal cavity; and backwards towards the fauces, impeding mastication, deglutition, speech, and respiration. At this stage of the disease, the countenance is most hideously disfigured, and the patient is an object well calculated to excite the deepest commiseration.

The integuments and mucous membrane are generally sound in the *earlier stages* of the malady; but after a certain period, varying from several months to a year or more, they begin to assume a livid hue, become deeply congested at one or more points, and at length yield to ulcerative action. The consequence is, a fungating and rapidly spreading sore, the seat of a thin, sanious, muco-purulent, or sanguinolent discharge, very abundant, excessively fœtid, and highly irritating in its character. Pure blood often proceeds from it; sometimes very small in quantity, at other times so copious as rapidly to undermine the strength, and bring on hectic irritation and exhausting night-sweats.

In the *latter stages* of the disease, sometimes before, but generally not until after ulceration has set in, the lymphatic glands of the temple, behind the ear, and under the jaw become enlarged and contaminated, and finally burst from over distention of their contents. The countenance assumes a peculiar cadaverous hue and expression; the patient rapidly loses flesh and strength; colliquative diarrhea and sweats supervene; the pain is excessive; and death, now a welcome visiter, closes the scene of agony and strife.

The *pain* attending this affection has no fixed or definite character. In general, it is dull and aching, not unlike the tooth-ache, to which, in fact, it is usually at first referred by the patient. As the tumor augments in volume, and encroaches upon the surrounding structures, the pain commonly becomes more severe and constant, and assumes a sharp, lancinating character. It is usually most violent at night, and in damp, cloudy states of the atmosphere; it is also greatly influenced by the condition of the stomach and bowels, by mental emotion, and by whatever has a tendency to produce derangement of the secretions. In some cases the pain is tensive and pulsatile, especially when the tumor is very large, or is about to become the seat of suppuration. Occasionally, again, it is of a neuralgic character, having, like an intermittent fever, regular paroxysms and intermissions. It is rarely limited to the diseased mass, but extends through the neighboring parts, and sometimes even to a considerable distance.

The *progress* of the tumor is variable; sometimes very rapid, at other times quite tardy. In 1844, a boy, five years of age, was brought to me for an affection of this kind, which caused his death in less than six months from its commencement. The tumor was of extraordinary volume, filling the mouth, throat, and nostrils, and causing the most frightful deformity. A short time before death, it gave way in the mouth, fungating and bleeding so profusely as rapidly to undermine the foundations of life.

A case of still more rapid termination was under my observation last summer, in a colored man, aged twenty-seven, belonging to a Mr. Lewell, of Palmyra, Tennessee. The disease, occupying the right maxillary bone, was first noticed in April, 1851, and by the 2nd of July, when the man arrived in town, it had already attained an enormous bulk. The tumor formed a large prominence on the cheek, and almost filled the mouth, rendering it very difficult for the patient to swallow, and to articulate so as to make himself intelligible. It rapidly increased in size, extending behind the ear, to the eye and temple, beyond the median line, and below the inferior maxillary bone, throwing the chin several inches over to the opposite side. In a few weeks after his arrival, a small slough formed upon the cheek, over the most prominent portion of the tumor, evidently from the effects of the pressure of the morbid mass, and this was soon followed by the appearance of a large fungus, which was the seat of a constant sanious and most loathsome discharge, attended, occasionally, with copious hemorrhage, and the most violent pain imaginable. Under the influence of this accumulation of evils, his general health, already much impaired by previous suffering, rapidly gave way, and on the 9th of August he died. Upon inspection, the tumor was found to be composed of a soft, fluctuating, brain-like substance, the true character of which it was impossible to mistake.

In this case, no one could, of course, for a moment, entertain the question of an operation; the period for such a procedure, if proper at any time, had already passed when the man reached Louisville; and the only reason why he was not sent back, at once, was that he was unable to bear the fatigues of the journey.

On the other hand, I have seen several cases in which the fatal event was delayed for several years. My experience is that the malady is usually more rapid here, as in other parts of the body, in children and youth than in the middle-aged and old.

When such a tumor is *examined*, after removal, it is found to exhibit all the characteristic features of encephaloid formations. That portion which occupies the antrum is commonly quite soft and pulpy, resembling, at least faintly, a section of the brain, both in its color and consistence. The osseous structure is generally broken down and disorganized, quite vascular, and so soft as to be easily cut with the knife. In some specimens it is entirely, or nearly entirely, absorbed; while, in others, it is replaced by fibro-cartilage, or cartilage, intermixed with spicula and scales, remnants of the original substance. In nearly all cases, the morbid mass is remarkably vascular, being pervaded, in every direction, by large vessels, the walls of which are exceedingly brittle, and therefore liable to yield under the slightest impulse. It is owing to this circumstance that these tumors frequently attain such an enormous volume, and that, when ulceration sets in, they are so liable to fungate and bleed.

The *recognition* of encephaloid disease of the superior jaw is usually not difficult. The rapid growth of the tumor, its steady encroachment upon the adjacent parts, its soft and elastic feel, the livid aspect of its buccal portion, and the sharp darting pains which attend it, readily distinguish it from all other formations of this division of the skeleton. In the latter stages of the affection, the fungous character of the ulcer, and the sanious, sanguinolent, and bloody discharges, together with the sallow and cadaverous state of the countenance, and the enlargement of the neighboring lymphatic ganglions, leave no doubt about its nature. Important information will also be furnished by the history of the case, and by the fact that encephaloid occurs at all periods of life, while some of the other maladies of this region are met with only at certain ages.

Where any doubt exists respecting the character of the tumor, no objection lies against the use of the exploring needle, which will at once inform us as to the consistence of the tumor, and the nature of its contents. If it is encysted in its character, an escape of serous, or muco-sanguineous fluid, will afford the necessary intelligence, and enable us to arrive at a pretty satisfactory conclusion in regard to the proper mode of procedure. Should encephaloid matter be present, the smallest quantity, in fact the merest particle, will, if subjected to the microscope, reveal the characteristic cancer-cell. I consider it of great importance that the exploring instrument should be as fine as possible, that the puncture which it makes may heal by the first intention, and not permit the protrusion of any of the contents of the tumor. There is much danger in malignant diseases, where this precaution is omitted, of doing harm by inducing premature ulceration, fungation, and hemorrhage, followed by the rapid destruction of the patient. I much fear that surgeons have paid too little attention to this circumstance.

Encephaloid of the jaw seldom *co-exists* with malignant disease in other parts of the body. The affection, in fact, in the great majority of instances, is more habitually local than when it invades the cellular tissue, eye, and glandular organs. It is, doubtless, owing to this circumstance that excision of the disease, especially in its early stage, is occasionally followed by a successful result; though, in general, as was before intimated, the prognosis is most unfavorable.

2.—The tumor next in point of frequency is the *fibro-cartilaginous*, or, as is it not uncommonly, but incorrectly termed, *sarcomatous*. It is not easy to describe this variety of growth, so diversified and multiform are its component elements. Its most ordinary form is the fibrous, in which, as the name implies, the fibrous structure predominates, though there is often, if, indeed, not constantly, an intermixture of other elements, especially the cartilaginous; small cysts, containing a thin serous, glairy, or sanguineous fluid, are sometimes interspersed through these tumors. Few vessels are apparent in their structure, and hence they seldom attain a very great bulk, or advance with much rapidity. For the same reason they do not bleed much when ulceration takes place, or during attempts at extirpation.

The original site of this tumor, like that of the encephaloid, is usually the antrum; but it also sometimes begins in the proper substance of the bone, in the socket of one of the teeth, usually in that of one of the grinders, in the periosteum, or between the periosteum and the outer surface of the bone. However this may be, the substance of the bone is gradually broken down and disintegrated, and ultimately lost in the new deposit.

The fibro-cartilaginous tumor of the upper jaw, notwithstanding that it is not, in general, very vascular, occasionally attains a very large size. Instances have been reported in which it was as big as a fœtal head. Usually, however, it is small, not exceeding the volume of an orange, or an ordinary fist. One of the most interesting facts connected with this tumor, is its non-malignant character, or its tendency not to return after extirpation.

The fibro-cartilaginous tumor can generally be *distinguished* from encephaloid and other formations of the upper jaw, first, by the slowness of its growth; secondly, by its globular, ovoidal, or botryoidal shape; thirdly, by its circumscribed character, or indisposition to ramify through the surrounding parts; fourthly, by its firm and unyielding feel; fifthly, by its painlessness; and sixthly, by the absence of contamination of the neighboring lymphatic glands. The tumor manifests little or no tendency to ulcerate, and the mucous-membrane of the mouth is of a more florid color than in encephaloid. There is also less serous and bloody discharge. The general health is not deteriorated, and the countenance is free from the peculiar sallow and dejected expression which forms so remarkable and characteristic a feature in malignant disease. The fibro-cartilaginous tumor is not confined to any particular period of life, but is most common in young adults.

3.—The *epuliform* tumor is occasionally seen upon the upper jaw, though its favorite site is the lower. This is a sarcomatous, or fibrous growth, of a soft spongoid consistence, and of a red florid complexion, springing originally from the gum, or the lining membrane of the socket of a tooth, and gradually extending to the bony texture, which, in time, is partially absorbed, or expanded into a soft, elastic, parchment-like shell. By and by, the teeth loosen and drop out; the morbid mass ulcerates and fungates; and the patient is constantly annoyed by a sanious, or bloody discharge, so offensive as to be loathsome both to himself and to his neighbors. The tumor seldom extends beyond the alveolar process; but occasionally it forces its way, even at an early period, into the maxillary sinus, rapidly filling it up, and then protruding its walls in every direction, as in the true osteocephalomatous formation. A decayed tooth, a blow upon the jaw, or injury upon the gum, are the most common causes of this affection. Not unfrequently, however, it comes on spontaneously, under the influence, apparently, of some constitutional taint.

The *diagnosis* of this form of tumor rarely offers any difficulty. Its exposed situation, its connection with the teeth and alveolar process, its want of sensibility, its florid appearance and spongoid consistence, are almost pathognomonic of the nature of the affection. It is only in the more advanced stages of the malady, when the body of the jaw has become extensively involved, when the surface of the tumor has begun to ulcerate and sprout, and the discharge is copious, bloody, and offensive, that the discrimination will be likely to prove embarrassing. The tumor, under such circumstances, might be easily mistaken for medullary sarcoma; a circumstance, however, which need not be particularly regretted, inasmuch as the curative measures are the same in both cases.

The *progress* of this morbid growth is sometimes remarkably rapid; at other times, slow and gradual. As it increases in volume, it must necessarily encroach very materially upon the mouth, thereby interfering with the tongue, teeth, and fauces. Its character is usually malignant, and hence, unless it is thoroughly extirpated, it is almost certain to return after removal. Like encephaloid, it rarely co-exists with malignant disease in other parts of the body. Indeed, it would seem, even in its worst forms, to partake more of the cancroid than of the true cancerous structure and habit.

4.—The *sero-cystic* tumor of the upper jaw is uncommon, and, fortunately, devoid of malignancy. Its usual site is the alveolar process, where it has been known to attain the volume of a hen's egg, and even of a large orange. It is composed of a thin, semi-transparent, or slightly opake bag, occupied by a colorless, or sanguineous fluid, or by a glairy, mucilaginous substance. Sometimes, though rarely, there are two such cysts, either closely connected together, or seperated by a kind of osseous septum. The bone around the tumor is expanded into a thin, elastic, crackling, parchment-like shell, and is easily penetrated by a sharp instrument, the puncture giving vent to the characteristic contents of the cyst. This, in fact, is the best diagnostic sign of the morbid growth. The general health remains unaffected in this disease, which is always tardy in its progress, and manifests no disposition to extend among the adjacent structures. Where any doubt exists as to the real nature of the case, a resort to the exploring needle will usually at once dispel it.

It is not often that this tumor calls for removal of the affected bone; in general, it will suffice to puncture it occasionally with a small trocar to evacuate its contents, the escape of which is often followed by the rapid contraction and final obliteration of the sac. Where there is a strong tendency to reaccumulation, a larger opening should be made, and tincture of iodine thrown in, to promote the object in view. It is only in old and intractable cases that excision of the affected bone will be likely to be necessary.

5.—Excision of the superior maxillary bone is sometimes required on account of the presence of an *exostosis.* The tumor, varying infinitely in regard to its size and form, is most common in young and middle aged subjects; it may appear upon any part of the bone, and gradually augmenting in volume, may at length involve it in its entire extent. It is a strictly local affection, the result often of external violence; and rarely, if ever, degenerates into malignant action. An exostosis is a true bone, growing, as the name implies, from bone, and composed of precisely the same antomical, chemical, and microscopical characters.

An exostosis is easily *recognized*. Its chief peculiarities are, its excessive hardness, its slow growth, its freedom from pain, the absence of disease in the surrounding structures, and the unimpaired state of the general health. There is no discharge of blood, or muco-purulent matter, no tendency to ulceration, no alteration in the skin of the face, or mucous membrane; the principal inconvenience which the patient experiences is from the size of the morbid growth, which is occasionally very considerable, and from its consequent interference with the functions of the adjacent parts. When doubt exists in regard to the real character of the disease, a small exploring needle, introduced at various points of the swelling, will at once decide the question.

6. Under the same head with exostosis may be mentioned an enlarged state of the bone, constituting a species of *hypertrophy*. An example of this variety of morbid action will be found in the first case appended to this paper, in which the tumor, occurring in a young lady of nineteen years, was occasioned by the irritation of an inverted, or misplaced canine tooth. The bone was expanded into a thin shell, about the volume of a large hickory nut, and was of a hard, firm consistence, with but little pain, and no constitutional derangement. The tumor was evidently of local origin, and the operation, performed in April, 1845, was perfectly successful; the part remaining well up to the present moment.

Mr. Stanley* mentions an instance in which the hypertrophy involved the whole jaw, which was removed by operation; the patient, however, a girl of fifteen years, dying on the tenth day from erysipelas. The cavity of the antrum was completely obliterated by the osseous matter, which exhibited all the characters of the natural texture. The general health was perfectly good, and no disease was discoverable in the surrounding parts. The hypertrophy had been in progress for eight years, and presented itself mainly in an oblong, hard, and painless enlargement of the ascending portion of the bone, projecting into the face, and extending into the nostril.

Such enlargements, it is obvious, are not malignant, and are, in general, easily distinguished from this class of affections by their slow development, their firm, unyielding consistence, their painless character, and the healthy condition of the adjacent parts.

7.—*Scirrhus* of the superior jaw must be exceedingly rare, as I have not, in a practice of nearly twenty-five years, met with a solitary case. Many instances of the kind are, I am aware, reported in our surgical and periodical literature; but it is perfectly clear to my mind that they were examples, not of scirrhus, but of encephaloid. Should this disease ever occur here, it would be likely to show itself in advanced life, as a hard, firm, solid tumor, slow in its progress, and characterized by sharp, lancinating pain. The growth would be likely to attain much less bulk than soft cancer; nor would it be so liable to fungate and bleed.

8.—*Melanosis* of the upper jaw is likewise very unfrequent, and no example of it has yet fallen under my observation. The same remark is applicable to colloid, which is, if possible, a still more rare disease than melanosis. A fatty tumor has been described as occurring in the upper jaw; and Gensoul gives an instance wherein

*Treatise on the Diseases of the Bones, p. 227; Phil: 1849.

the antrum was occupied by an erectile growth. The tumor, in this case, was remarkably soft, and so vascular that it appeared, after having been injected with quicksilver, to be composed chiefly of minute vessels.

9. *Polypoid* tumors of the antrum are occasionally seen, but much less frequently than is generally supposed. Indeed, they must be exceedingly rare. They are benign or malignant, generally the latter; are difficult to diagnosticate; and are always prone to return after extirpation. These tumors require no particular description; because they do not, in the great majority of instances, differ from the medullary formation, from which, in fact, it is usually impossible to distinguish them.

It would seem that *exsection* of the upper jaw was practised by Acoluthus as early as 1693. The operation was repeated, in the eighteenth century, by Planque, David, and Beaupreau, and, early in the present century, by Siebold, Deschamps and Klein.* In all these instances, however, it was limited to the removal of a portion of the bone. In Great Britain, extirpation of the upper jaw does not appear to have been practised until 1826, when it was executed by Mr. Lizars, of Edinburgh, who, I believe, is fully entitled to the merit of having first suggested the possibility and advantage of removing the *entire* bone, when the seat of disease.† Three years prior to this period our countryman, Dr. Alexander H. Stevens, then Professor of Surgery in the University of New York, excised nearly the whole of the upper jaw, along with portions of the malar, ethmoid, and sphenoid bones, for a fungous tumor of the antrum.‡ In May, 1824, Dr.

*Velpeau's Operative Surgery, 2. p. 728.

†Liston's paper on Tumors of the Mouth, in London Medico-Chirur. Trans. 20, p. 178.

‡Cooper's Surgical Dictionary, Appendix by Dr. Reese.

David L. Rogers, of New York, performed a similar, but still more formidable operation, exsecting the superior maxillary bones, anterior to the first molar tooth on each side, together with the two inferior turbinated bones, a part of the nasal septum, the vomer, and a portion of the right antrum.* Since the period here adverted to, the operation in question has been repeatedly executed by American surgeons, among whom McClellan, Mott, Mussey, Mütter, Pancoast, and the two Warrens, deserve special mention. I am not aware that it has been performed by any one in Kentucky, except myself.

Previously to undertaking any operation for the removal of the upper jaw, whether for a part or the whole of it, a careful inquiry should be instituted into the history of every case, with a view of ascertaining its particular character, the condition of the adjacent parts, and, above all, the state of the general system. If the tumor be of the cephalomatous kind, large in size, rapid in its development, dipping into the surrounding structures, and attended with contamination of the neighboring absorbent glands, failure of the general health, and a sallow cadaverous countenance, all manual interference is out of the question. To operate, under such circumstances, would only hasten the patient's fate, and tend to bring surgery into discredit. On the other hand, however, there is no reason, in my opinion, for refraining from the use of the knife, even in this form of disease, provided the morbid growth is comparatively small and tardy in its progress, and the surrounding parts and constitution are perfectly sound. It is a well ascertained fact, as was previously stated, that encephaloid of the upper jaw rarely co exists with malignant disease in other organs; and, hence, if such a tumor be exsected at an early period of its development

---

*Surgical Essays, p. 81, New York, 1848.

while its action is strictly local, success must occasionally crown our efforts, at least so far as to prolong the patient's life, if nothing more, beyond the period he could otherwise attain. The propriety of such a course is fully sanctioned by the experience of the best surgeons. In the case of Mrs. Green, the third on my list, although the disease for which the bone was removed was unquestionably of an encephaloid nature, and has repeatedly returned, five years have elapsed since the first operation, but for which she must have perished long ago. The great fault, in regard to the operation, is, that it is generally not resorted to sufficiently early in the disease, owing to the ignorance of the patient, and his unwillingness to submit to the knife as long as he can persuade himself that he may be relieved by ordinary means. The case being thus trifled with, the surgeon, when it falls into his hands, finds himself in the disagreeable dilemma of being obliged to decline all interference, or to attempt an operation, the result of which is almost sure to be unsatisfactory. He has to contend with science and self-interest, on the one hand, and sympathy for his patient, on the other; and his decision will be influenced by the predominance of the one or the other. Most men, if not wholly governed by selfish considerations, would be likely, if the case is not altogether desperate, to afford the sufferer a chance for his life. I do not wish to be understood, in making this remark, that I am an advocate for the indiscriminate use of the knife; by no means; on the contrary, as is well known to my friends and pupils, no one more constantly and loudly condemns a reckless and unprincipled resort to instruments. I merely wish to say, and I say it boldly, that I abhor timid surgery, and that cautious, calculating conduct in operators which induces them to pay more regard to their own reputation than to the welfare of their patient; operators who would not hesitate to consign the poor sufferer to his cruel fate, because they are afraid of public opinion. I do not envy such men their feelings; I despise them as much, and as cordially, as I despise the merest knives-men. It should never be forgotten that a case, which may appear hopeless in the eyes of one surgeon, may not appear so in the eyes of another, equally enlightened; and that a case, which one surgeon has condemned as unsuitable for an operation, has sometimes been easily relieved by another equally expert. It cannot be denied that surgery has achieved some of its proudest triumphs in this manner.

In non-malignant tumors of the upper jaw, no fears need be entertained about a relapse; the only difficulty here is about the diagnosis. When this has been clearly established, and the patient is in a proper condition for an operation, the sooner it is performed the better.

The *manner* in which the operation is to be performed, must necessarily vary with the circumstances of each individual case. When the tumor is of considerable size, requiring sometime for its removal, the patient should always be placed recumbent, with a broad and rather thin pillow under the head and shoulders, and the face inclining to the opposite side. Very few persons, whatever may be their courage and fortitude, can bear the shock and fatigue of an operation of this kind while sitting up. The recumbent position is more particularly necessary when chloroform is administered. I find considerable objection expressed by certain gentlemen against the use of this article in operations upon the mouth; but without, it seems to me, any just reason; at any rate, I have resorted to this agent ever since its introduction into the materia medica in every severe case of amputation, both of the upper and lower jaw, that has come under my observation, and I have, thus far, had no cause to regret the practice. The only objection that can be urged against anæsthetics in these operations is, that the patient, being unconscious, is unable to clear his mouth of blood; but if proper care be taken to compress the vessels as soon as they are divided, and the head be placed upon a low pillow, no danger can ensue from this source; I certainly have never witnessed any, and until I do I shall not hesitate to continue the practice, whatever others may say and do.

I have never found it necessary, in any of my operations upon the upper and lower jaw, to secure the carotid artery, as a means of preventing hemorrhage. Indeed, one cannot but be surprised that such a procedure should ever have been recommended, much less employed, by any sensible surgeon. My experience is that there are no organs in the body, of the same extent in their natural and diseased condition, the removal of which is attended with so little hemorrhage. I am not in the habit even of employing compression of the carotid artery in these operations; and, as to tying that vessel as a means of security against the loss of blood, I should as soon think of ligating the femoral artery for the same purpose. Nothing could be more absurd and unnecessary. The chief danger from bleeding is in the subcutaneous arteries, especially the facial and its branches, and expert assistants should always be at hand to seize and compress them as soon as they are divided. I rarely, if ever, stop to tie a vessel during any operation, however extensive, or complicated. My experience has taught me that there is, in general, no necessity for such a course, which is always attended with vexatious delay and annoyance. The deep seated arteries, involved in tumors of the upper jaw, seldom bleed much, if care be taken to keep beyond the limits of the diseased structures. If this precaution be neglected, the hemorrhage may be copious, and even exhausting. The oozing which takes place from the osseous surface, after exsection has been effected, generally speedily ceases of its own accord from the contact of the atmosphere; where this is not the case, a stop may usually be easily put to it by the application of compresses, wet with a saturated solution of alum. The actual cautery could be necessary, in such a case only, when a portion of the tumor has been left behind; a circumstance which ought never to happen in the hands of any one, as it must necessarily lead to a speedy reproduction of the mischief.

The direction, extent, and number of the *incisions* through the soft parts must, of necessity, vary with the situation and extent of the tumor. In all these respects, much must be left, in each case, to the judgment and experience of the operator. When the morbid growth is comparatively limited, and seated upon the anterior, or antero-lateral aspect of the jaw, we shall generally be able to dispense with external incisions altogether, as our object may be readily enough accomplished simply by dissecting off the lip from its attachments, and holding it out of the way with the finger, or blunt hook. The surface of the tumor having been thus thoroughly denuded, the bone is to be attacked with the saw, and severed fairly beyond the line of disease. By this procedure, which is admirably adapted to the more simple forms of morbid growth, the operation is deprived of much of its severity, and followed by no deformity of the features, except what may result from the caving in of the integuments.

When the tumor involves the body of the bone, and is of considerable bulk, as, for example, as big as a fist, the plan which I usually adopt, and which has always answered my purpose most fully, is to make one long, curvilinear incision, extending across the most prominent part of the tumor, from the commissure of the lips towards the zygomatic process of the malar bone, terminating within a few lines, half an inch, or an inch of the external angle of the eye, according to the volume of the abnormal mass. In this manner are formed two flaps, the upper of which is convex, and the lower concave, which are then carefully dissected up by bold and rapid strokes of the knife, and held out of the way by trust-worthy assistants, who at the same time take care to compress the bleeding vessels. The space which this procedure affords is, in general, quite sufficient for the easy removal of the entire tumor, however large, or extensive its connections. In my own cases, it has always answered my purpose most thoroughly. Should it, however, be inadequate, it can be easily increased to the requisite extent by a horizontal cut along the inferior border of the orbit, as far as the nose. In making the first of these incisions, the facial artery is necessarily divided, and in the second, the superior maxillary nerve, together with many of the branches of the portio dura of the seventh pair. In consequence of the injury thus sustained, the parts supplied by these nerves remain a long time paralyzed, though ultimately the face regains, in great degree, its accustomed power and expression.

Some surgeons recommend two incisions under the above circumstances; but such a procedure is not only unnecessary as it respects the facility of excision of the tumor, but exceedingly objectionable on account of the greater deformity to which it must lead.

When the tumor, or enlargement, occupies the anterior and upper portion of the jaw, the external incision may extend vertically upwards, by the side of the nose, from the free border of the lip to a level with the orbit of the eye. This will enable the operator to detach the ala of the nose, and to remove, if necessary, the ascending process of the jaw bone, the ungual bone, the inferior turbinated bone, and even the vomer.

When the antrum is mainly implicated in the disease, two incisions, representing the form of an inverted L, are necessary, the vertical limb corresponding with the ascending process of the maxillary bone, and the horizontal one with the inferior border of the orbit of the eye.

Whatever be the form and direction of the incisions, care should be taken that they are always sufficiently extensive to afford ready access to the diseased mass. Nothing can be more embarrassing, or display greater want of judgment in the operator, than a want of room in a case of this kind.

The necessary incisions having been made through the soft parts, and the flaps properly dissected up, the next step in the operation is to remove the tumor. As a preliminary measure, one or more teeth must be extracted, to make room for the play of the saw and other instruments. This part of the operation ought always to be performed as soon as the patient is fairly under the influence of chloroform, prior to the division of the soft structures. Performed after that, it is liable to occasion delay and annoyance.

The separation of the jaw is usually the work of a few minutes. The limits of the disease being generally well defined, care must be taken to keep on the outside of them, for the double purpose of avoiding hemorrhage, and removing the whole of the morbid structure. The best contrivance for executing this part of the operation is a saw, the blade of which is about three inches long, eight lines in width, and a little rounded off at the end, with sharp, wide-set teeth. It should be provided with a stout handle. The surgeon should be furnished with a series of instruments of this kind, as one is rarely sufficient. He should also have several chisels, a pair of pliers, a lenticular, and a stout scalpel, with a piece of steel at the extremity of the handle, which should be bevelled off, that it may be used as a scraper and a cutter, as may be deemed necessary. Mr. Liston has declaimed quite eloquently against the chisel and in favor of the pliers in this operation; probably, because the latter instrument was his own invention, and one of his well-known hobbies! I have used it on two occasions upon the upper jaw, but it failed so utterly of its purpose, and produced such a terrible noise and crash that I never think of it without horror. The chisel may have its faults; it may jar and shake the head; but I should be loth to believe that it could possibly jar and bruise the parts as much as a pair of the best pliers, however carefully and gently managed.

When it is designed to remove the whole jaw, the saw should be carried, successively, through the alveolar process in front, and the horizontal plate behind, close to the middle line, as far back as the corresponding portion of the palate bone; the mucous membrane of the roof of the mouth having been previously divided with the scalpel, to prevent it from being torn and bruised, as it otherwise would be. Next the saw is to be applied to the malar bone, at or near its junction with the maxillary, and finally to the nasal process, which is generally divided on a level with the lower margin of the orbit of the eye. The orbitar plate of the jaw bone is commonly left intact, as it does not often participate in the morbid action. Should it do so, it should be cautiously removed with the chisel and knife, lest the eye and its appendages sustain mischief. All that now remains to be done, is to sever the tumor at its junction with the pterygeid process and palate bone; and here, again, the chisel and knife will come in excellent play. The main tumor having been removed, the parts are carefully sponged, and any remnants of diseased substance cleared away with the lenticular and other suitable instruments. If a particle of the new deposit be suffered to remain, the operation will prove a failure, as it will be certain to be soon followed by a reproduction of the tumor.

The next step is to sponge away the blood, and to search for, and tie any vessels that may seem inclined to bleed. It is seldom, in any case, that more than two or three ligatures will be required. To arrest the oozing of blood from the deep portion of the wound, and give support to the cheek, I am in the habit constantly of stuffing the osseous gap with patent lint, wet with a saturated solution of alum. The edges of the cutaneous wound are then approximated by twisted sutures and collodion strips; and a compress being applied upon the cheek, the parts are supported by a roller, passed round the head and under the chin in the form of a figure 8.

The *after treatment* must be conducted upon strictly antiphlogistic principles; the head and shoulders are maintained in an elevated position and as quiet as possible; light diet and cooling drinks are enjoined; the bowels are moved by aperients; and the secretions are corrected, if necessary, with calomel, or blue mass. The needles are removed at the end of the fourth day, when the edges of the incision will generally be found perfectly united. The great danger after this operation is erysipelas of the face and head, which commonly shows itself within the first forty-eight hours, and should be promptly met by the usual remedies. Several of my cases suffered severely from this cause. Few patients die from the shock of the operation. The subject of my sixth case died from pneumonia nearly three weeks after the removal of the whole of the upper jaw, together with the inferior turbinated and a portion of the malar and palate bones.

The patient soon becomes accustomed to his loss; and the function of deglutition, at first so difficult and annoying, is gradually performed with nearly its original facility. Even the faculty of mastication is regained much more rapidly than one, unacquainted with the compensating powers of nature, might be lead to suppose. The deformity of the face is often comparatively trifling; and the defect in the mouth may usually be remedied, in the more favorable cases, by artificial means. It is surprising how much, even in a short time, the cavern contracts and diminishes, and how all the surrounding and associated parts accommodate themselves to their new situation.

It would be interesting to give an account of the *re-*

*sults* of the different operations that have been performed for the removal of the upper jaw in malignant and other diseases; but for such an undertaking we have, unfortunately, no precise data, nor could such data be obtained without a most careful and elaborate analysis of all the reported cases; a task for which I have neither leisure nor inclination. When the malady is of the encephaloid character, it may be safely assumed that it will return, sooner or later, in almost every instance, no matter how thoroughly the abnormal structure may have been extirpated. In the non-malignant varieties of tumor, on the contrary, there is no reason to apprehend a relapse, any more than in the same class of affections in other parts of the body. Of eleven cases operated on by Lizars, Syme, Robert, Scott, Earle, Guthrie, and Hetling, only one was completely successful. Gensoul has removed the upper jaw four times, with one case of failure, and in that the affection was of a malignant character. Dieffenbach reports five instances, all successful; and the late Mr. Liston, seven, in only one of which the disease returned, and proved fatal. Rignoli has one successful case, and one failure.*

Looking at my own cases, I find that four have died; three from a recurrence of the disease, and one from the effects of pneumonia, nearly three weeks after the operation; that two have entirely recovered; and that one has suffered a relapse but is still living, five years after the extirpation of the morbid growth. In this case, that of Mrs. Green, the third on the list, I excised nearly the whole of the jaw in 1847, and since then several pieces have been removed, at different intervals, by Dr. Sloan, of New-Albany. Although the tumor, in this instance, was not inspected with the microscope, yet I am certain, from a

---

*British and Foreign Medical Review, 7. 250.

careful examination of its physical properties, that it was cephalomatous in its character; a circumstance which imparts more than ordinary interest to the case, inasmuch as it holds out great encouragement to the surgeon in an affection usually regarded as desperate, if not hopeless. In a case of brain-like tumor of the upper jaw, reported by Dieffenbach, the patient was subjected to five different operations before a final cure was effected.

CASES OF EXCISION OF THE UPPER JAW.

I shall close these remarks with a history of seven cases of excision of the upper jaw, which have been drawn up with great care, and the results of which have been satisfactorily ascertained in every instance.

Case 1.—The following case is remarkable chiefly on account of the cause of the tumor, which was quite small. Miss C., aged twenty-one, of Frankfort, Kentucky, came under my observation in June, 1843, for an enlargement of the left upper jaw-bone, which she first noticed about two years and a half previously; it occupied the alveolar process, was free from pain and discoloration, and produced no appreciable prominence on the corresponding side of the face until eighteen months ago. About six months after the tumor first attracted attention, the first bicuspid tooth became loose, and was extracted by a dentist; the operation was followed by considerable hemorrhage, but this soon ceased, and the parts healed in a short time. After this, the tumor increased a little more rapidly, and by degrees the second bicuspid became loose; but, as it did not ache, it was not removed.

At the time of Miss C.'s visit, the tumor was about the volume of a large hickory nut, and seemed to be formed at the expense mainly of the outer surface of the alveolar process; it was hard and inelastic, devoid of pain or soreness, and unaccompanied by any disease of the gum. The general health was, and, in fact, always had been, excellent.

As the tumor was making steady, though slow progress, and was productive of some deformity of the lip, as well as of great mental disquietude, I determined at once to remove it. As a preliminary measure, the canine tooth was extracted by my friend, Dr. Somerby. The bone was then sawed through, at the site of this tooth, as far as the inner table of the alveolar process, and to the same extent at the posterior limit of the tumor, which was then raised with a chisel and a strong knife, the lip having been previously detached from its upper extremity. As the internal lamella of the bone was perfectly sound, it was, of course, permitted to remain, especially when it was ascertained that the whole trouble had been occasioned by the presence of a tooth, apparently a cuspid, a little smaller than natural, but well formed, with the crown reversed, or directed upwards towards the antrum of Highmore. The parts soon healed after the operation, and the patient recovered with hardly any defect, except what resulted from the extraction of the tooth at the time of the operation. Could the real nature of the tumor have been determined before the operation, this tooth would, of course, have been saved.

Supernumerary teeth are not of unfrequent occurrence, especially in the upper-jaw; they are much more common among the permanent than the temporary set; and their shape generally resembles the cuspid or canine teeth. They are not, however, confined to the front of the jaw, though they are more frequent here than elsewhere. Inverted teeth are seldom met with. Dr. Somerby and Dr. Goddard, two eminent and experienced dentists of this city, inform me that they have never met with an example. Mr. Hunter* gives a drawing of the upper jaw of a child, in which the cuspid tooth was inverted, "so that its

*Natural History of the Teeth, Tab. 8, Fig. 9.

point was turned up against the jaw, and the growing mouth of its cavity towards the gum." Albinus relates a singular instance where a tooth was found on each side in the ascending process of the maxillary bone, between the nose and the orbit; it was long and very thick, and situated in such a manner that the crown looked upwards towards the eye. No satisfactory explanation has yet been furnished of the manner in which this vicious direction of the teeth is brought about. The subject is interesting in connection with that of osseous tumors of the superior maxillary bone.

Case 2.—Miss B., aged nineteen, of a florid complexion, and rather delicate health, from the vicinity of Shelbyville, Kentucky, observed, nine months ago, several weeks after a severe attack of bilious fever, a small tumor in the right upper jaw, which gradually increased in size, and at length formed a slight prominence under the cheek. At her visit to me in April, 1845, the parts exhibited the following appearances: The tumor, which was firm and solid, projected internally a little beyond the middle line, while at the side it extended from the anterior edge of the lateral incisor to the horizontal plate of the palate bone. The alveolar process was much expanded, and the middle grinder, having become loose, had been extracted by the family physician, under the belief that the disease was an abscess of the antrum of Highmore. The other teeth were sound, and firm in their sockets. The integuments of the cheek and the mucous membrane of the mouth were healthy. The speech and deglutition seemed to be little, if at all, affected. The tumor was the seat of slight, darting pains, which had latterly become more constant and severe, and its volume had nearly doubled itself during the last eight or nine weeks. The general health had not materially suffered.

Having properly prepared the system, I performed the operation of excision of the affected parts on the 19th of

April, 1845, in the presence of Drs. Johnston, Cochran, Somerby, Colescott, and T. L. Caldwell, the latter three of whom afforded me their kind assistance. The patient being a woman of unusual fortitude and determination, sat upon a chair, with her head reclining against the breast of one of the gentlemen just named. As a preliminary step, Dr. Somerby extracted the lateral incisor and last molar tooth. An incision was then made over the prominent portion of the swelling, beginning at the commissure of the mouth, and terminating near the outer angle of the eye, its direction being slightly curvilinear. The two flaps being next detached, and held out of the way, the jaw was divided, with a Heys' saw, by the side of the middle incisor, from whence the instrument was carried through the horizontal plate, close along the raphe, as far back as the palate bone. The tumor was then attacked with the chisel and mallet by cutting through the outer wall of the antrum, the cavity of which was occupied by a soft fungous mass, and was evidently the original seat of the whole mischief. This being thoroughly cleared out with the lenticular and handle of the scalpel, the operation was completed by sawing the jaw at its junction with the malar and palate bones. An artery, a branch of the one which supplies the vomer, was cut, and required torsion. No vessels were tied. The quantity of blood lost did not exceed four or five ounces. The woman bore the operation admirably.

The cavity left by the removal of the tumor was filled with patent lint, and the wound closed with four twisted sutures, aided by a compress and roller. As there was a good deal of pain, a grain of morphia was administered after the girl went to bed.

Everything went on well until the fourth day, when erysipelas, which had been for sometime epidemic in the city, made its appearance at the upper angle of the wound, and gradually extended over the whole face, ears, and forehead. Under the influence of a few active mercurial cathartics and the application of the tincture of iodine, the disease was soon arrested, and comfort restored to the part and system. The sutures were removed on the fourth day, when the union was found to be complete.

As soon as suppuration was fully established in the bony cavity of the mouth, the lint was removed, and after the parts were well syringed with a solution of chloride of soda, it was replaced, to afford support to the cheek. These dressings were renewed daily for upwards of a week.

Miss B. left Louisville in a fortnight after the operation, almost as well as ever, and with hardly any perceptible scar, so perfect was the union of the wound. There was, of course, a considerable retrocession of the cheek, and great impediment in articulation; the bottom of the osseous cavity was covered with healthy granulations, and there was still some discharge of pus.

The tumor was carefully examined after removal, and exhibited all the characteristic features of encephaloid. Indeed, no morbid growth that I have ever inspected was better marked in this respect.

I saw this young lady four years after the operation, and am happy to say that she is still living, her residence being the neighborhood of Paducah. Her articulation is greatly improved, and the deformity of the face is so slight as to escape casual observation.

Case 3.—Mrs. Susan Green, aged thirty-eight, a married woman, the mother of nine children, from Crawford county, Indiana, visited Louisville in June, 1847, to consult me respecting a tumor of the left superior maxillary bone, which had of late become a source of much distress to her. Her statement was that the affection was first noticed in 1830, as a small, movable lump, situated opposite the second large grinder, between the median line and the alveolar process. The growth was slow and gradual until about four years ago, when, after cauterizing it, for the third time, with nitrate of silver, it began to advance very rapidly, and has continued so until the present time. The tumor is now as big as an ordinary fist, and produces great distortion of the features; protruding the ball of the eye, pushing out the cheek, and closing the left nasal cavity. Internally it overlaps the right raphe of the roof of the mouth, while posteriorly it projects slightly beyond the maxillo-palatine suture, displacing the tongue, and thrusting it over towards the right side. Mastication and deglutition are both considerably impeded. The tumor is hard, firm, and inelastic. Last winter, the two bicuspid and two anterior molar teeth became loose, and were extracted, without causing any particular pain, or serious hemorrhage.

Until about six months ago, the tumor was free from suffering, except that it was the seat, pretty constantly, of a feeling of tension or tightness. Since then it has become affected with a dull aching pain, which radiates about in different directions, and is particularly severe in the left malar bone, and corresponding nostril. Mrs. Green is not aware that her jaw has ever received any injury, and she knows of no cause of the disease. Until a few months age, her health was always remarkably good; of late, she has lost flesh, and her countenance has assumed a rather sallow aspect.

Excision of the tumor was performed on the 15th of June, with the aid of my pupils, Dr. Summers, Dr. Bozeman, and Dr. Ector, and in the presence of a number of physicians and students. Dr. Goddard had the kindness to extract, as a preliminary measure, the canine and last molar teeth. The patient being placed upon a table, with her head resting upon a pillow, and being fully under the influence of ether, an incision was made over the prominent portion of the tumor, from the left angle of the mouth to within a short distance of the outer canthus of the eye, in a slightly curvilinear direction, the convexity of the curve looking downwards. The flaps being dissected off, the saw was applied to the anterior and lateral part of the jaw, from whence it was carried obliquely backwards through the raphe of the horizontal lamella of the maxillary bones to a little beyond their junction with the palate bones. Continuing the operation, I next divided, with the saw and pliers, the root of the ascending process of the bone, nearly on a level with the floor of the orbit, and finally the malar process and a part of the horizontal plate of the palate bone. Having thus severed its principal and most important connections, the tumor was wrenched from its bed with the hand and chisel, and the bottom and sides of the chasm immediately cleared of every thing betraying the slightest appearance of disease. A good deal of oozing of blood took place at this stage of the procedure, but this soon ceased spontaneously, and did not afterwards recur.

The osseous cavity being stuffed with lint, wet with a saturated solution of alum, the edges of the wound were brought together by five twisted sutures, and the woman, somewhat exhausted by the operation, removed to her bed. Not more than five or six ounces of blood were lost during the operation, which occupied but a few minutes. No arteries, except a small muscular branch, required the ligature. Some vomiting occurred at the close of the operation, from the effects, apparently, of the ether. As soon as this had passed off, a grain of morphia was given, to allay pain, which was now considerable, and mustard was applied to the chest and extremities.

It is not necessary to present a diary of the symptoms and treatment of this case, which progressed most favorably, with the exception of a slight attack of erysipelas of the face, from the time of the operation till the patient left Kentucky, nearly three weeks after. The sutures were withdrawn at the end of the fourth day, when the wound was found to have united, in its whole extent, by the first intention. The lint was removed from the mouth as soon as the suppurative process had fairly begun, and was re-inserted daily for a week, when its employment appearing no longer necessary, it was dispensed with. As a detergent, free use was made of the chloride of lime in solution. When the patient went home, the bottom of the chasm was covered with fine healthy granulations, and nearly all the discharge of pus had ceased. The face was but slightly disfigured. The voice was, of course, materially changed, and there were many words which she was unable to pronounce with distinctness.

Having learned that Mrs. Green had suffered a relapse, and that she had become a patient of Dr. Sloan, of New Albany, I addressed a note to that gentleman, who kindly furnished me with the following facts: "Mrs. Green called upon me on the 4th of March, 1849, with a considerable enlargement of the median portion of the alveolar process of the left upper jaw, which I removed the next day, by making a vertical incision from below the inner angle of the eye to the border of the left lip, detaching the ala and septum of the nose, and then cutting the bone obliquely across from between the incisors on the right side, through the nasal cavity, to the opening made in your operation. The piece thus removed contained, I think, the roots of the canine and two left incisor teeth, with the central incisor of the right side.* About eight months after, she applied to me for a small growth on the posterior boundary of the cavity, which I removed with its bony attachments.

I saw Mrs. Green again in May, 1852. Her disease had

---

*Dr. Sloan is, I think, in error in saying that the piece included the canine tooth, as I have a distinct recollection that I sawed the bone through the socket of that tooth, in my operation.

again returned, involving the posterior wall of the antrum, the palatine arch, and the pterygoid process, and extending towards the coronoid process of the lower jaw. I expect she will visit me next autumn."

Case 4.—William Collinridge, aged sixty-six, a brass-founder by occupation, and a married man, of temperate habits, and nervo-sanguineous temperament, was recommended to me by his professional adviser, Dr. Hagan, of this city, in the latter part of July, 1847, on account of a tumor in the right upper-jaw. He had always been healthy, until about four months ago, when he became feeble and nervous, and soon after discovered a slight swelling of the gum, attended with aching of the large grinders. His suffering increasing, he consulted a physician, who advised the extraction of the affected teeth, which was accordingly done, but with only temporary relief. The operation was attended with considerable hemorrhage, and was soon followed by an increase of the swelling, and a recurrence of the pain, in all its previous violence.

When Collinridge first consulted me, the tumor was so large as to form quite a prominence under the cheek, and to encroach a good deal upon the buccal cavity; his speech was thick and indistinct, and he was unable to masticate solid food. The enlargement extended from the lateral incisor to the posterior extremity of the bone, and had a softish and elastic feel, not unlike an encephaloid tumor. The mucous investment was remarkably florid and congested, but sound in texture, and the integuments of the face were perfectly healthy. The pains were excessive, and almost without intermission; darting about in different directions, and usually most severe at night and during damp and cloudy states of the weather. All the teeth on the right side, excepting the two incisors, had either dropped out or been extracted.

On the 9th of August, 1847, assisted by Dr. Hagan and several of my private pupils, I excised the diseased mass, the patient having previously inhaled ether. The incision was made, as in the cases already described, from the commissure of the lips in a slightly curved direction, to within a short distance of the external angle of the eye. The flaps being dissected up, and held out of the way, the saw was applied in front, close by the central incisor, and thence carried back to the palate bone. The bone was then cut at its junction with the malar bone, at the ascending process, and along the orbitar plate, when the whole mass was forcd out of its bed. A considerable flow of blood took place, at this stage of the operation, which had to be stopped for a few minutes, until the patient recovered from the effects of the resulting exhaustion. A large sponge was immediately pressed upon the bleeding surface; and as soon as I was able to proceed, the blood was wiped away, and the osseous cavity cleared of its morbid contents with the knife, chisel, and lenticular. The hole was then stuffed with lint, wet with a saturated solution of alum, and the edges of the wound were approximated by a compress and bandage. Only two small arterial branches required the ligature.

Nothing of any special interest occurred after the operation, which was not followed by a single bad symptom. The wound united by the first intention, and the sutures were removed at the end of the fifth day. Gradually granulations sprung up at the bottom of the gap, greatly diminishing its depth and extent, and thus aiding in the recovery of the power of speech and deglutition. The generel health rapidly improved, and the patient was perfectly free from the local suffering which had so much harrassed and distressed him before the operation. Things went on in this manner for about six weeks, when, all at once, a small tubercle made its appearance upon the skin, at the former site of the tumor, and soon acquired the size of a twenty-five cent piece; it was hard and slightly elastic to the touch, projected some distance above the surrounding surface, was of a dark livid hue, and the seat of a constant sharp, shooting pain. No attempt was made to remove this, for it was but too evident that it was of a malignant character, and that any further operation would be worse than useless, the more especially as the general health now began to give way, and the granulations at the bottom of the wound presented a pale, flabby, and unnatural aspect. Soon after this, small tubercles, of a kind similar to that just mentioned, showed themselves upon the temple, rapidly increasing in size, and greatly aggravating the local distress. The pains were excessive, shooting and darting about in every direction, through the face and head, and requiring the constant use of morphia for their subjugation. The tumor on the cheek acquired a large bulk, and at length ulcerated, discharging a large quantity of sanguinolent fluid, and occasionally pure blood. Excessive emaciation supervened, followed by hectic irritation, and colliquative diarrhœa, under which the patient sunk in October, 1848, fourteen months after the operation. No examination was made.

The morbid mass, in this case, possessed all the external features of encephaloid. The bone was deprived of its normal texture, and the soft portion of the tumor was of the color and consistence of the adult brain.

Case 5.—The subject of this case was Sylvia, a negress, married, thirty-five years of age, and a servant of Mr. Stephen Brown, of Springfield, Kentucky. She came under my care in 1849.

The disease first appeared about three years ago, as a boil, on the inside of the gum, and has gradually but steadily attained its present dimensions. During the last few months, however, its growth has been much more rapid than before, and this, so far as can be ascertained, without any assignable cause. The tumor, at this time, embraces the jaw, from the right canine tooth to the malar articulation, filling up the zygomatic fossa, and hideously distorting the features. In front, it does not quite reach the middle line, but posteriorly it projects beyond the raphe, and extends as far back as the soft palate, which, together with the side of the pharynx, is red and tumid, causing much difficulty of articulation and deglutition. The largest portion of the tumor, however, is external to the jaw, within the lips and cheek, reaching from the zygomatic process of the temporal bone to the angle of the inferior maxillary bone behind, and to the anterior portion of the mouth in front. In its shape it is irregularly globular, its longest diameter, the antero-posterior, being about three inches and a half. Of a pearly pink appearance on the inside, it is of a deep red, almost purple color, externally beneath the cheek, and is traversed by numerous vessels, filled with dark blood. In its consistence it is hard and inelastic, except along the groove formed by the pressure of the teeth of the lower-jaw, where it is somewhat soft, and ulcerated. It has been more or less painful ever since its commencement, especially at the back part. Much distress has also been experienced, of late, in the forehead, temples, and nape of the neck.

During the progress of the tumor, the back molar tooth became loose, and was extracted, showing a small abscess filled with purulent matter. The operation afforded only temporary and partial relief, and was attended with a good deal of hemorrhage. Subsequently, the other grinders shared the same fate. A neighboring physician, supposing that the main disease was in the antrum, punctured it with a lancet, but nothing but blood issued from the wound.

About three months before her arrival in Louisville, a hard, circumscribed swelling, as I am informed by Dr. Hughes, of Springfield, appeared on the front of the neck, just above the sternum, slightly compressing the trachea, and impeding respiration. After sometime, fluctuation was perceived, when it was opened, and soon after it gradually went away, except a little remnant, which is still discharging a thin, sanious fluid.

Although my patient's health was a good deal impaired, and the tumor was of an unpromising character, I considered it my duty to operate, being satisfied that, bad as the chances were for recovery, the case was not wholly hopeless. Accordingly, on the 12th of April, assisted by Drs. Colescott, T. G. Richardson, Henry, and Thomson, the woman was placed upon the table, and put under the influence of chloroform, the external lateral incisor having been previously extracted by Dr. Goddard. The external incision, curvilinear in its direction, extended from the commissure of the lips to within a few lines of the outer angle of the eye, over the most prominent portion of the morbid mass, the surface of which was fully exposed by the reflection of the two flaps of integuments. The bone being divided in front and internally, with a pair of sharp pliers, and the anterior wall of the antrum laid open with a stout knife, the tumor was turned out of its bed, followed instantly by a copious gush of blood, and the fainting of the patient. Pulling the pillow away from her head, and thrusting a sponge into the bottom of the chasm, I scraped out, with all possible haste, the remnants of soft structure, and then sawed the malar and palate bones; finishing the operation by dissecting out a process of the tumor which had dipped in between the arch of the palate and the pterygoid muscles, between the upper and lower jaw.

Two small arteries being secured, the blood was sponged away, and the osseous cavity stuffed with lint, wet with a saturated solution of alum. The edges of the wound were approximated by four twisted sutures and collodion plasters, and the patient, much revived by means of hartshorn and brandy, put to bed. A grain of morphia was administered soon after the operation, and until the following morning the woman was most assidiously watched by my assistants, Drs. Thomson and Henry, whose kindness I had frequently witnessed on similar occasions. Complete reaction had taken place by 4 o'clock in the afternoon, four hours after the operation, and the night was passed in a comparatively tranquil and comfortable manner, there being but little pain and difficulty of deglutition. The pulse was eighty and soft; there was little thirst, and the woman had taken some gruel. The face and eyelids were slightly swollen.

On the 14th, two days after the operation, the patient had several free motions from the effect of an ounce of oil taken the previous evening; she had slept some, and there was no fever or thirst. The swelling of the face and eyelids was the same as on the 13th. On the 15th, the patient expressed herself quite comfortable, and the parts looked well. On the 16th, I withdrew the needles, and removed the lint from the mouth. The next day, the fifth from the operation, the collodion plasters came off, thus exposing the wound, which was found completely united by the first intention.

After this period no diary was kept; the case went on well; and, in less than three weeks, the woman returned to her master, rapidly improving in strength and flesh. Sometime afterwards, as I learn from Dr. Montgomery, of Springfield, the disease reappeared in the roof of the mouth, in the form of a small excrescence, which rapidly increased until it attained the volume of a pullet's egg. It was attended with severe pain in the head, and great swelling of the nose, which looked as if it were distended with a large polypus. The corresponding eye participated in the disease, and finally burst. There was great difficulty of breathing; and, about four weeks before the woman died, the disease showed itself in the forehead and opposite eye. Death took place on the 15th of December, 1850, twenty months after the operation.

Case 6.—Benjamin Hudson, of Alabama, aged fifty-two, a farmer, married, and the father of eleven children, of a sanguineo-bilious temperament, dark complexion, and temperate habits, was always healthy until the latter part of November, 1850, when he perceived, without any assignable cause, a small circumscribed swelling in the right cheek, a short distance below the outer corner of the eye, and just within the malar bone. The face was sore and tender to the touch, but the skin was free from discoloration. About three weeks after he observed this swelling, he noticed, for the first time, a little tumor, about the size of the end of a finger, hard and firm in its consistence, situated under the cheek in contact with the gum. In fact, it looked and felt like a gum-boil. It bled upon the slightest touch; indeed, sometimes spontaneously; now slightly, and then quite profusely. Increasing gradually but steadily in volume, it at length encroached upon, and loosened the anterior and posterior molar teeth, so as to render it necessary to extract them in February, 1851; the middle one having been removed about eighteen years previously, along with two small fragments of the jaw-bone. By the middle of April, the tumor had attained the volume of a goose's egg, and was making most rapid progress. The pain which attended it was of a sharp, lancinating character, and was so violent and incessant as to deprive the patient almost entirely of appetite and sleep. He had also by this time lost a great deal of flesh and strength.

The patient reached Louisville on the 3d of May, 1851, in the following condition. The right cheek was occupied by an enormous tumor, of an elongated ovoidal shape, being longer in the antero-posterior than in the vertical diameter; it was very tender on pressure, and the seat of a severe and constant pain, sharp and darting in its character. The integuments were of a purple color, and the corresponding portion of the upper lip was very much swollen and distorted. The tumor extended up to the eye, backwards over the zygomatic process, forwards to within a few lines of the nose, and downwards towards the base of the lower-jaw. Viewed within the month, it was found to reach inwards to the middle line, forwards to the lateral incisor, backwards to the soft palate, and downwards into the floor of the buccal cavity. It was of a pale reddish complexion, firm and elastic, tender on pressure, and irregular in its shape. Towards its anterior and lateral aspects, where it lay in contact with the cheek, was an ulcer, about the size of an American eagle, the surface of which was of a brownish color, rough, and fungous. It was free from pain and hemorrhage, and the patient was unconscious of its existence. The tumor seriously impeded articulation and deglutition, but the respiration was natural. The patient had no appetite, and he had not slept well since he left home; he was thin, and his countenance, although not sallow, had a haggard, care-worn expression.

Having purged and dieted my patient for nearly a week, I operated upon him in the presence of Drs. Hardin, Raphael, Thomson, Richardson, and other medical gentlemen. Chloroform was given him to the requisite extent; and, as a preliminary step, Dr. Goddard extracted the central incisor. By means of a curvilinear incision, extending from the commissure of the lips to the zygomatic process, an inch and a half from the outer canthus of the eye, two flaps were marked out, which were then dissected up, and carefully held out of the way. The jaw being sawed through in front at the middle line, the instrument was then carried through the horizontal plate of the *left* jaw bone, close along the raphe, and back through the entire length of the corresponding portion of the palate bone. The next step of the operation consisted in dividing the maxillary process of the malar bone, the periosteum of which appeared to be involved in the disease. The nasal process of the jaw bone being now sawed, just below the lacrymal sac, the morbid mass was attacked with the chisel and mallet, and severed along with the orbitar plate, and the horizontal portion of the palate bone. The inferior turbinated bone, and a portion of the nasal septum were also detached. Finally, the operation, one of the most horrid I have ever executed, was completed by freeing the surface of the osseous cavity, with its various chasms and ramifications, of all diseased matter, with the knife, scoop, and lenticular.

The patient towards the close of the operation became faint, and was obliged to use cordials and other restoratives. The loss of blood did not exceed eight or ten ounces; only a few little arteries required to be secured. The bony cavity was stuffed with lint, wet with alum water, and the parts were approximated with six twisted sutures and collodion strips, supported by a compress and bandage.

As soon as the effects of the chloroform had passed off, the patient took a grain of morphia. In the evening I learned that he had been quite comfortable nearly all the afternoon; he had slept some, and had little or no thirst, nor much local uneasiness. The following night was passed in tolerable comfort; next day he had some fever; and he complained of a sense of tension in the deep portion of the wound. On the third day, slight erysipelas came on in the right side of the face, and rapidly increasing in extent, soon spread over the forehead and temple. Recovering from this disease, in a short time, every thing went on well for a fortnight, when the patient was seized with pneumonia, which gradually progressed from bad to worse, despite the means employed for arresting it, until the 28th of May, when it proved fatal. It is proper to observe that the wound healed nearly in its entire extent by the first intention.

An examination after death revealed high inflammation of the lungs and pleura on both sides, with considerable effusion into the thoracic cavity. No carcinomatous disease was found any where, and the parts concerned in the operation exhibited a perfectly healthy aspect.

Case 7.—In the following case, which came under my observation in May, 1851, only a portion of the upper jaw was removed, the disease necessitating the operation having apparently commenced in the maxillary sinus.

J. H. P., of Henderson county, Kentucky, aged fifty-eight, a tall, slender, hard-working farmer, had his attention arrested in the early part of February, 1851, by a watering of the left eye, followed, soon after, by a severe, dull aching pain, confined, at first, entirely to that organ, but subsequently diffused over the upper part of the face, the forehead, and temple. He had always enjoyed good health up to this time, and was not aware that he had received any local injury. About the middle of April, he first noticed a swelling in the lower part of the orbit, and also a free discharge of muco-purulent matter from the left nostril.

Mr. P. arrived in Louisville, accompanied by his physician, Dr. Howard, of Owensboro, on the 24th of May. The upper portion of the left side of the face was, at this period, affected with a tumor, which encroached so much upon the corresponding eye as to protrude it partially from its socket, and which was the seat of a sharp, lancinating pain, darting about in different directions, and so constant and intense as to deprive him, in a great measure, of appetite and sleep. The eye had a watery, inflamed appearance. The integuments of the face were in a sound state. Upon inspecting the left nostril, I found it occupied by a large swelling, evidently a portion of the tumor already described, and discharging a large quantity of thin bloody fluid, of the most fœtid character. The general health was somewhat impaired, but the patient was in good spirits, and anxious for an operation.

Although I must confess that I did not like some of the symptoms of this case, and so expressed myself to Dr. Howard, yet such was the desire of the patient to be relieved, that I was at length prevailed upon to undertake an operation. Accordingly, having dieted him for a few days, and given him some purgative medicines, I proceeded to my task on Tuesday, the 27th of May, aided by my friends, Dr. T. G. Richardson, Dr. Thomson, Dr. Hardin, and Dr. Raphael. Chloroform having been administered, two incisions were made at right angles with each other, in the form of an inverted L, the limb of one extending along the side of the nose as low down as the lip, and that of the other along the margin of the orbit of the eye. The flap was then dissected up, and the wall of the antrum attacked with the saw, chisel, and lenticular, until the whole of the diseased structures, osseous and soft, was turned out. A process of the tumor dipped back deep into the orbit, and was removed with much difficulty, owing to the importance of the surrounding parts, and the manner in which they were obscured by the bleeding. The whole of the upper jaw, excepting the alveolar and horizontal portions, was cut away along with a part of the malar bone. A large artery, a branch of the internal maxillary, bled profusely at the bottom of the osseous cavity, and was finally, after much ado, secured with a ligature. In consequence of the great oozing of blood, it was necessary, before replacing the integuments, to stuff the cavity with lint, wet with a strong solution of alum. The edges of the flap were approximated with three interrupted sutures and collodion strips, supported by a compress and bandage.

The patient was put in bed, his head reclining upon a high pillow, and immediately took a grain of morphia. A short time after he lay down, blood was seen escaping from the nostril; but the quantity was small, and the discharge soon stopped of its own accord, and did not subsequently recur. At the end of the fifth day, free suppuration being established, the lint was carefully withdrawn through the nose, and through a small opening in the upper limb of the wound, the greater portion of which had united by the first intention. The cavity was afterwards syringed daily with a weak solution of chloride of soda, under the use of which, together with mild tonics and a nourishing diet, the parts rapidly healed, and the general health greatly improved. The patient left Louisville on the 8th of June, with directions to pay the utmost attention to himself in every particular. Within a few days after he reached home, he was seized with a violent attack of dysentery, which was then epidemic in that region of country, and under which he sunk, completely exhausted, on the 2nd of July. Dr. Howard, of Owensboro, from whom I have learned these particulars, in a letter dated August 17, 1852, informs me that a tumor, about the volume of a medium-sized marble, and the seat of the most excruciating pain, appeared, about a week before he died, in the left orbit, just above the inner canthus.

I was not aware, until I received the above intelligence, that Mr. P. had died so soon after he left Louisville, and from a cause so entirely foreign to that of the disease for which I operated upon him. How long he might have lived, had he not been thus assailed, after the affection of the orbit showed itself, is a matter which must be left wholly to conjecture. Deducting this case from those which are stated, under the head of *results*, as having proved fatal, it will be perceived that only two died from the *immediate* effects of a relapse of the malady for which recourse was had to the knife.

It is proper to add that the tumor, in this case, exhibited all the physical and microscopical characters of encephaloid, as I was assured by my friend, Dr. T. G. Richardson, who carefully examined it within a few hours after its removal.

I had forgotten, until after the greater portion of this paper was printed, that Dr. Stevens, to whom, in common with some of my brethren, I have ascribed the credit of being the first to excise the upper jaw in this country, had been anticipated in this undertaking by Dr. Jameson, of Baltimore. In looking, accidentally, over the fourth volume of the American Medical Recorder, I find that this distingnished surgeon performed the operation in question in November, 1820, nearly three years prior to that of Dr. Stevens. The primitive carotid was tied as a precautionary measure, and the patient remained well when the case was reported, nearly three months after the operation. The account of the case is accompanied by a graphic engraving, showing the size and situation of the tumor, as well as the great deformity to which it gave rise.

Louisville, Aug. 24, 1852.

